# Digital Literacy and International Students’ Subjective Well-Being: The Mediating Role of Psychological Resilience and Positive Affective Engagement

**DOI:** 10.3390/bs16101722

**Published:** 2026-09-22

**Authors:** Shaojun Ma, Xin Li, Yuanyuan Wang, Jin Zhang

**Affiliations:** 1 School of International Education, Tianjin University, Tianjin 300072, China; mashaojun0212@tju.edu.cn; 2 College of Management and Economics, Tianjin University, Tianjin 300072, China; 3 School of Education, Tianjin University, Tianjin 300350, China; zhangjin@tju.edu.cn

**Keywords:** international students, digital literacy, subjective well-being, psychological resilience, positive affective engagement

## Abstract

In the global digital revolution, digital literacy has emerged as a critical competency that influences the academic progress and social integration of international students, as well as significantly impacting their subjective well-being in host countries. This study employed a questionnaire survey among 455 international students in China and used structural equation modeling and STATA to examine the impact of digital literacy on international students’ subjective well-being and its underlying mechanisms. Empirical results revealed that digital literacy had a significant positive direct effect on subjective well-being (*β* = 0.425, *p* < 0.001). Psychological resilience mediated this relationship (indirect effect = 0.307, S.E. = 0.046, 95% CI = [0.216, 0.397]), and positive affective engagement also served as a mediator (indirect effect = 0.129, S.E. = 0.053, 95% CI = [0.023, 0.230]). These findings indicate that digital literacy not only directly enhances international students’ well-being but also operates through two indirect pathways: by fostering resilience and by stimulating positive affective experiences in creative learning and community co-construction. This study elevates digital literacy from a “tool skill” to “survival capital” within the antecedent model of Chinese international students’ well-being, offering a new perspective and analytical framework for cross-cultural adaptation research in the digital age. Furthermore, it uncovers the dual pathways through which digital literacy affects international students’ subjective well-being, deepening the understanding of cross-cultural adaptability in the digital era, and carrying significant theoretical value and practical implications.

## 1. Introduction

The deepening development of the Fourth Industrial Revolution has allowed digital technologies to permeate social life, reshaping human learning, work, social interactions, and lifestyles (OECD, 2019; UNESCO IITE, 2022). Against a backdrop of globalization and technological change, the internationalization of higher education is advancing at an unprecedented pace (Al-Hattami, 2025; UNESCO, 2023). An increasing number of international students are choosing to study in countries with different cultures (S. Ma et al., 2025a). As well as facing academic challenges, they need to adapt to entirely new social and technological environments. This process of cross-cultural adaptation can lead to personal growth and psychological well-being (Feng & Sumettikoon, 2024). For international students in foreign countries, the digital environment serves as a vital channel for acquiring knowledge and completing academic work. It is also a key tool for maintaining connections with home societies and building social support networks in the host country. Therefore, international students have become an integral part of the global digital citizenry (L. L. Chen et al., 2021; Gu et al., 2023). Consequently, the digital literacy (DL) of international students has evolved from a supplementary skill to a core competency impacting their academic development, social integration, and personal well-being.

Digital literacy(DL) encompasses far more than just traditional computer operating skills (Chang & Kuo, 2025; C. Liu et al., 2025). It encompasses understanding, evaluating, utilizing, creating, and disseminating digital content, and engaging safely and responsibly in digital environments (Ji & Li, 2025; Nagel et al., 2023; Zheng et al., 2025). However, the 2024 International Perspectives on Digital Literacy report indicates that learners globally still lack sufficient development of higher-order digital literacy competencies in education (Fraillon, 2025). China has prioritized enhancing overall digital competence by issuing multiple policy documents, such as the Action Plan for Enhancing Digital Literacy and Skills Among All Citizens, and implementing key initiatives in recent years. For international students in China, DL enables effective use of online libraries, academic databases, online courses (e.g., MOOCs), and collaborative software. This helps them to overcome academic barriers caused by language difficulties and other obstacles, thereby enhancing their learning efficiency and academic confidence (Nami & Asadnia, 2024; Wang et al., 2024). In everyday life, the digital competencies developed through DL help international students to solve practical problems such as getting around and shopping, reduce loneliness caused by homesickness and cultural differences, and speed up their integration into local society (S. Ma et al., 2025b). DL thus forms the “cultural map” on which international students rely to survive and develop within China’s digital landscape.

Despite the growing importance of DL in global student learning, the ways in which it influences international students’ subjective well-being (SWB) remain largely unexplored. SWB is defined as an individual’s overall cognitive and emotional evaluation of their quality of life (Diener et al., 2018a, 2018b). International students may experience challenges such as culture shock, language barriers, identity crises, social isolation, and uncertainty about the future, all of which could potentially threaten their SWB (Arani et al., 2017; Cao & Meng, 2022). Numerous studies have confirmed that a higher level of subjective well-being is positively correlated with superior academic performance, greater learning engagement, stronger environmental adaptability, and distinct personality traits (Anglim et al., 2020; Bücker et al., 2018; Wong et al., 2024). Consequently, it is a crucial psychological indicator of the success of their study abroad experience. Current attempts to establish links between digital literacy and well-being predominantly focus on specific populations or contexts. Some studies have examined the impact of digital literacy on well-being among older adults (Hänninen & Tiihonen, 2026; Hofer et al., 2019; Hu et al., 2025; Zhan et al., 2025), while others analyze how educators and students develop digital literacy and pedagogical skills in the digital age (Cattaneo et al., 2022; Chiu et al., 2022; Y. Liu et al., 2024; Yang et al., 2025). However, most previous evidence comes from non-cross-cultural contexts, and direct empirical tests are still lacking as to whether digital literacy can significantly enhance international students’ subjective well-being under the pressure of an unfamiliar culture. The process of improving digital literacy may also entail short-term frustrations that lower the fulfillment of students’ psychological needs (Laaber et al., 2024; Valkenburg et al., 2022). Some studies further indicate that digital engagement on social media can trigger upward social comparison in virtual spaces, potentially even generating negative affect (Ding et al., 2025; Pang, 2021; Verduyn et al., 2020). Nevertheless, international students are “double strangers” in that they simultaneously experience cultural distance and rapid changes in the digital environment, so the relationship between their digital literacy and well-being may possess unique dynamics that have not been captured.

Based on this, drawing on the unique cross-cultural background and learning experiences of international students, this study incorporates both psychological resilience (PR) and positive affective engagement (PAE) into a unified analytical framework, and proposes and tests a theoretical model of how digital literacy affects international students’ subjective well-being. The contributions are twofold. First, as a capacity to recover and grow from adversity, PR serves as a protective resource for international students in coping with cultural shocks and academic setbacks. By framing digital literacy as a builder of resilience, this study highlights a theoretical novelty—shifting the understanding of digital tools from mere “functional use” to “psychological capital transformation.” Second, PAE moves beyond the traditional focus on cognitive learning engagement, concentrating instead on the positive experiences stimulated by creative learning and community co-construction in digital environments. This emphasizes that subjective well-being stems not only from “problem solving” but also from “meaning making,” thereby broadening the value dimension of digital literacy research. The simultaneous inclusion of both mediators is not a simple additive exercise, but rests on the theoretical premise of their functional specialization in cross-cultural adaptation—namely, “instrumental adaptation” (via PR) versus “affective adaptation” (via PAE).

Therefore, this study primarily addresses the following research questions: (1) As a core competence for international students in the digital era, does digital literacy have a significant positive direct effect on their subjective well-being? (2) Does PR mediate the relationship between digital literacy and international students’ subjective well-being? (3) Does PAE mediate the relationship between digital literacy and international students’ subjective well-being? To this end, drawing on two core psychological resource perspectives, this study examines the mediating roles of PR and PAE, respectively, in the process by which digital literacy influences subjective well-being.

The remainder of this study is structured as follows: Section 2 presents the literature review and research hypotheses; Section 3 outlines the methodology, including information on the sample and the research methods employed; Section 4 reports the findings and discusses model fit, direct and indirect effects, and offers further interpretations of the results; Section 5 concludes by summarizing the findings and identifying limitations.

## 2. Literature Review and Hypothesis

### 2.1. Direct Association of DL on SWB

Digital literacy (DL) emphasizes individuals’ higher-order ability to access, understand, evaluate, create, and communicate information in digital environments. Not only does it help international students to overcome language and cultural barriers, it also enhances their self-directed learning abilities and sense of social integration (Koşar, 2024; L. Ma, 2025). As a key indicator of quality of life, subjective well-being (SWB) is boosted by DL, enhancing international students’ self-efficacy and life satisfaction (Feng, 2024) and promoting their well-being.

Firstly, the vast amount of information and convenient services offered by digital platforms can help international students to overcome the practical difficulties they encounter when arriving in a foreign country. DL is, in essence, a critical resource. International students in a foreign environment are subjected to multiple pressures, including cultural distance, language barriers, and information asymmetry, which may lead to the continuous depletion of their resources. Using digital tools to establish social connections and create/share digital content gives them a sense of accomplishment and self-efficacy. This reduces anxiety stemming from uncertainty and enhances their sense of control over their lives and overall satisfaction (S. Ma et al., 2025b; Zhang & Hyland, 2025). Secondly, enhanced DL empowers international students with greater autonomy and control. Those with high DL can utilize online resources for academic research more effectively, access course information and overcome learning challenges (Vaszkun & Mihalkovné Szakács, 2025; Prior et al., 2016). Digital literacy, as a resource-generating capacity, can help international students effectively acquire informational resources (e.g., academic databases, living guides), instrumental resources (e.g., online translation tools, navigation software), and social resources (e.g., online communities, alumni networks). Individuals with abundant resources are less vulnerable to stress shocks and can form a positive cycle through resource reinvestment, thereby maintaining higher levels of academic and life satisfaction (Wu, 2025). Thirdly, DL impacts international students’ social connections and emotional expression within digital spaces (Boie et al., 2024). Digital media are vital tools that enable international students to maintain connections with their home countries during transnational studies. They provide indispensable channels for sustaining emotional bonds with family and friends, and for accessing emotional support. Maintaining social connections is a key factor in enhancing international students’ SWB and strengthening their sense of belonging and security (S. Ma et al., 2025b). Finally, when confronted with vast amounts of information and digital resources, international students with high DL can discern the authenticity of information, thereby reducing unnecessary worry and distress. Accurate digital judgment is crucial when handling complex information related to visas, academics and daily life. It helps individuals avoid potential risks, enhancing their sense of security and quality of life (Al-Obaydi et al., 2025; Jusmawati et al., 2023). Effective digital safety literacy enhances their sense of security in the digital realm, mitigates negative experiences and promotes subjective well-being (Getenet et al., 2024; Pan et al., 2024).

Therefore, DL is a comprehensive digital competency that enables international students to maximize the advantages of digital environments while minimizing their potential risks. This has a positive and direct impact on their SWB. Based on this, the following hypothesis is proposed:

**H1.**  *International students’ DL is significantly positively associated with their SWB.*

### 2.2. The Mediating Role of PR

Psychological resilience (PR) is defined as an individual’s ability to adapt their coping strategies and maintain psychological equilibrium when facing adversity, trauma, tragedy, setbacks or major stressful events. It is also a dynamic process of recovery (Cui et al., 2023). For international students, PR enables them to cope with culture shock, overcome academic challenges, manage interpersonal conflicts and ultimately achieve personal growth and development (X. Chen et al., 2025). Among international students experiencing cross-cultural adaptation and high academic stress, PR is regarded as a core competency that safeguards their mental health and well-being (Hedeshi & Ghanizadeh, 2025). To this end, this study elucidates the mechanism through three pathways: cognitive empowerment, resource empowerment, and meaning empowerment.

Firstly, the cognitive empowerment pathway. PR endows digitally literate international students with stronger problem-solving abilities (Lam & Zhou, 2022). When facing complex or unfamiliar situations, digitally literate international students are more inclined to use digital tools for information retrieval, problem decomposition, and solution comparison (X. Chen et al., 2018; Solhi et al., 2025). The accumulated experience of such “digital problem-solving” reinforces individuals’ positive appraisal of their own coping capacity, thereby enhancing self-efficacy. According to social cognitive theory, high self-efficacy is a crucial cognitive foundation of PR, enabling individuals to interpret stressful situations as “manageable challenges” rather than “uncontrollable threats.” This shift in cognitive appraisal helps reduce anxiety and helplessness, which in turn is associated with higher subjective well-being. Secondly, the resource empowerment pathway. Digital literacy enables international students to transcend geographical and institutional barriers, gaining convenient access to online psychological support resources, such as online counseling, peer support communities for international students, and mental health applications (G. Li, 2025). Resilience theory posits that the availability of external support resources is a significant determinant of an individual’s level of resilience. The abundant support resources in the digital environment allow international students to restore emotional balance more quickly after psychological shocks, effectively buffering the erosion of well-being caused by acculturative stress. These digital resources provide immediate, accessible, and relatively private channels of support when individuals encounter acute stressful events. Finally, the meaning empowerment pathway. Digital literacy enables international students to actively engage with multicultural content—such as other students’ experience-sharing blogs, inspirational stories, and cross-cultural dialog videos—which helps them reinterpret the difficulties they encounter, reframing “exam failure” as “a necessary part of the learning curve” and “social setbacks” as “opportunities for enhancing intercultural competence” (Hou et al., 2022). This capacity for cognitive reappraisal is a core component of PR, enabling individuals to construct meaning and maintain hope in adversity, thereby sustaining their long-term well-being experience.

Therefore, digital literacy is positively associated with PR by strengthening individuals’ problem-solving beliefs (cognitive empowerment), expanding accessible psychological support resources (resource empowerment), and facilitating constructive cognitive reappraisal (meaning empowerment). As a protective psychological capital, PR is in turn positively associated with subjective well-being. On this basis, we propose the following hypothesis:

**H2.**  *PR mediates the relationship between international students’ DL and SWB.*

### 2.3. The Mediating Role of PAE

Positive affective engagement (PAE) refers to the positive emotions that individuals experience when participating in an activity. It encompasses emotional-level pleasurable experiences, cognitive-level deep thinking and behavioral-level active involvement (Fredrickson, 2001; Luque-Reca et al., 2022; Zhang et al., 2021). In this study, PAE refers to the immediate, context-bound positive affective states that individuals experience while using digital technologies to participate in specific learning, social, or cultural activities, with pleasure, excitement, interest, and involvement as its core characteristics. It is worth noting that PAE also differs from general positive affect, cognitive engagement, behavioral engagement, creativity, and concentration. General positive affect is one manifestation of PAE; cognitive and behavioral engagement emphasize attention, effort, and behavioral participation; creativity emphasizes the production of novel and useful ideas or works; and concentration emphasizes the focusing of attentional resources. This study argues that digital literacy can empower international students to transform potentially tedious, passive or solitary activities into enjoyable, interactive and meaningful experiences that stimulate PAE, thereby enhancing their subjective well-being. This is manifested in the following ways:

Firstly, DL transforms passive learning into active enquiry and creation, thereby sparking interest in learning. Traditional learning models can sometimes be one-sided and tedious. Students enrolled internationally with high DL transcend the limitations of teacher-delivered content, making the learning process more vivid and personalized. They use advanced DL to discover cultural experiences in virtual settings, such as exploring Dunhuang online to gain an understanding of China, and use digital technology to fuel their curiosity and thirst for knowledge (Dewaele et al., 2018; S. Ma et al., 2025a). This learning mode more readily obtains positive feedback from instructors, and the sense of achievement, pride, and pleasure experienced in this process are themselves affective experiences that belong to PAE, rather than creativity or cognitive engagement. Creativity is a cognitive and behavioral manifestation during the activity, whereas PAE is the affective experience that accompanies this process and further enhances international students’ well-being (S. Li et al., 2024; Vainio & Daukantaitė, 2016). Secondly, DL transforms passive waiting into active construction, thereby fostering a sense of belonging and pleasure. Enhanced digital communication and collaboration skills enable international students to establish and maintain positive interpersonal relationships more effectively; strong relationships are a vital source of positive emotions (Zou et al., 2018). Online learning platforms (e.g., Tencent Meeting) facilitate the formation of study groups with peers, enabling collaboration on projects and discussion of academic perspectives. This allows students to experience a sense of belonging and accomplishment through cooperation (Järvelä et al., 2016; Zhang et al., 2021). Students can also maintain connections with new friends via social media, deepening mutual understanding and emotional bonds. For example, international students organize weekend outings through online platforms such as WeChat, exploring urban landscapes with peers from diverse cultural backgrounds and fostering deep friendships through shared experiences. Such social interactions involving positive affective engagement bring a sense of belonging and joy; they are an important manifestation of PAE and a core driver for enhancing subjective well-being (Jin, 2018; Lee & Lee, 2021). Furthermore, DL transforms bystanders into active participants, deepening cultural understanding and appreciation. Cross-cultural adaptation extends beyond merely recognizing differences to encompass direct engagement and experience. DL provides international students with more diverse and accessible social entry points, such as writing vivid blog posts or creating engaging short videos, to document and share their remarkable experiences and personal growth abroad. This “immersive” cultural experience, enabled by digital media, is full of the joys of exploration, hands-on engagement and interaction. When such content receives likes, comments and positive feedback, it boosts international students’ self-worth. The resulting novelty, excitement and sense of belonging embody PAE, enriching their experience of studying abroad and elevating their overall life satisfaction.

In summary, PAE leverages digital technologies to make learning activities more exploratory and creative, social activities more proactive and community-oriented, and cultural activities more immersive and participatory (Aryani & Rosyid, 2025). It is worth noting that PAE is distinct from SWB. SWB refers to an individual’s overall cognitive evaluation of their quality of life and long-term affective balance (Diener et al., 2018a, 2018b), encompassing life satisfaction judgments and the ratio of positive to negative emotions over time. PAE, in contrast, focuses on transient positive affective responses within specific activity contexts, characterized by situational boundedness and temporal immediacy. This distinction is analogous to the difference between “the pleasure experienced during a meal” (PAE) and “overall satisfaction with one’s life as a whole” (SWB). DL, by empowering students’ autonomous creation, social connection, and cultural exploration in specific contexts, elicits immediate, context-bound PAE; the frequent experience of PAE, through the broaden-and-build and cumulative effects of positive emotions, is ultimately positively associated with SWB. Based on this, the following research hypothesis is proposed:

**H3.**  *PAE mediates the relationship between international students’ DL and their SWB.*

## 3. Method

### 3.1. Instrument Development

Based on the literature review and hypotheses, the focus of this study’s research question is on the experiential perceptions of international students at the perceptual level. A questionnaire method is employed for data collection. The questionnaire comprises four main sections. The first section is the Digital Literacy Scale for International Students. The second section is the Subjective Well-being Scale for International Students. The third section is the Mechanism Scale for International Students, which includes the Psychological Resilience Scale and the Positive Affective Engagement Scale. The final section collects participants’ basic information (i.e., control variables). This study uses Likert scales to assess all variables. Following Brislin’s (1970) methodology, the questionnaire was translated from English to Chinese to ensure clarity. Responses were collected using a bilingual format, presenting both the Chinese and English versions of the scales simultaneously. After discrepancies were identified, certain items were modified and adjusted to guarantee the study’s accuracy. Due to the specific context of the research, some scale items were modified and adjusted to accommodate the cross-cultural setting.

Digital literacy (DL). DL serves as the independent variable. Based on the UNESCO Digital Literacy Global Framework and S. Ma et al. (2025b), this study adapted a digital literacy scale for international students. This scale serves as the independent variable and comprises five dimensions: information and data literacy, communication and collaboration, digital content creation, safety, and digital learning. In measuring this scale, because the five dimensions are highly coexistent, intertwined, and function jointly in international students’ DL, and this study focuses more on the overall impact of digital literacy as a holistic capability on international students’ SWB rather than on isolated effects, this study constructed digital literacy by taking the arithmetic mean of the five dimensions, comprising 18 items in total. It uses a five-point Likert scale, where 1 = Strongly Disagree and 5 = Strongly Agree. Higher scores indicate greater digital literacy. Sample items include: “I can quickly, accurately and comprehensively retrieve the required course resources from the internet or databases”, and “I can effectively evaluate the reliability and quality of the content of online information.”

Subjective well-being(SWB). SWB serves as the dependent variable in this study. Drawing on the subjective well-being scales developed by Campbell (1976), Peng et al. (2026), and Zhang et al. (2026), this study adopted a measure comprising two dimensions: the Index of General Affect and life satisfaction. The variable consists of nine items and was originally administered on a 7-point Likert scale. Since all other scales in this study used a 5-point Likert format, the data from this scale were subjected to a standard linear transformation, with the converted 5-point score calculated as = [(original 7-point score − 1) × (4/6)] + 1. Higher scores indicate a stronger subjective sense of well-being. Sample items include “interesting,” “valuable,” “hopeful,” “rewarding,” and “life has been good to me.”

Psychological resilience (PR). In this study, PR serves as the mechanism variable. The Connor and Davidson Psychological Resilience Scale was used, employing a five-point Likert scale comprising 10 items. Responses ranged from 1 = Strongly Disagree to 5 = Strongly Agree. Higher scores indicate greater PR. Sample items include: “I can handle difficulties when they arise”, “My experiences have made me stronger”, “I recover quickly after illness or hardship”, and “I can concentrate on problems when under pressure”.

Positive affective engagement (PAE). PAE serves as the mechanism variable in this study. Adapted from Watson et al. (1988) and Joshanloo (2017), the Positive Affective Engagement Scale uses a 5-point Likert scale with 10 items. Scores range from 1 = Strongly Disagree to 5 = Strongly Agree. Representative items include: “I am interested in using digital technologies to engage with coursework”, “Innovations in digital technology excite me and stimulate my creativity”, and “I can maintain high levels of focus when using complex digital tools”.

Control variables. Demographic characteristics serve as control variables in this study. Since demographic traits may affect international students’ DL, typical statistical variables were controlled for, including gender, age, educational background and subject. Additionally, due to the specificity of the subject, the duration of residence in China and the length of Chinese language study were also controlled.

### 3.2. Participants and Procedure

This study focuses on international students with academic experience in China. International students in China are categorized as either degree-seeking or non-degree (e.g., foundation program) students, with the former encompassing undergraduate, master’s and doctoral levels. The sample of this study consisted primarily of international students from Tianjin University. Data collection was conducted in three phases from October 2024 to April 2025. In the first phase, non-probability sampling was used, with questionnaires distributed to course instructors across various schools, international student administrative staff, and through free distribution. In the second phase, snowball sampling was employed to purposively expand the sample through recommendations from international student dormitory managers and peers of existing participants, recruiting participants via peer referrals and course participation. In the third phase, based on the same theme as the graduation projects of international students supervised by the authors, a new round of data collection on international students’ perceptions was conducted. A total of 683 questionnaires were collected in this survey. The inclusion criteria for data samples were as follows: (1) holding a foreign passport and non-Chinese nationality; (2) being a formally registered international student (including degree-seeking and non-degree students); (3) having resided in China for at least one month. The exclusion criteria were as follows: (1) visiting students on short-term exchange programs (less than one month); (2) completing the questionnaire too quickly (less than 3 min); (3) selecting the same option for more than 80% of the items in the entire questionnaire; (4) duplicate IP addresses. Based on these criteria, a total of 455 valid questionnaires were obtained, yielding an overall valid response rate of 66.6% (see Table 1 for details).

### 3.3. Data Analysis and Modeling

This study uses SPSS 26.0, AMOS 22.0 and STATA 18.0 software primarily for data analysis. SPSS is primarily used to examine the reliability of the questionnaire scales. The Cronbach’s alpha values are 0.936 for digital literacy, 0.846 for subjective well-being, 0.892 for psychological resilience, and 0.935 for positive affective engagement. The selection of different software packages was driven by their respective analytical strengths. AMOS provides more comprehensive fit indices in structural equation modeling; STATA’s regression module facilitates the output of standardized results and multicollinearity diagnostics (VIF); and the PROCESS macro is specifically designed for mediation and moderation effects, automatically generating bootstrap confidence intervals. The use of the four software packages is not redundant but rather a division of labor based on their respective technical advantages. Accordingly, in this study, AMOS and PROCESS were primarily used to analyze model fit and test mediation effects, while multiple linear regression in STATA was mainly employed to examine the direct and mediation effects.

The framework illustrated in Figure 1 was established based on the literature review.

## 4. Results and Discussion

### 4.1. Assessment of the Model Fit

To mitigate the impact of common method bias, this study used an anonymous survey to minimize the effect of social desirability bias. However, the data may still exhibit some degree of common method bias. The single-factor test method proposed by Harman was adopted and exploratory factor analysis was conducted on all variables collectively. The first principal component, obtained without rotation, accounted for 39.088% of the variance, which is within an acceptable range and indicates that common method bias did not affect this study.

Using AMOS 27.0, factor analysis was conducted on four factors—DL, PR, PAE and SWB—as the basic model. The results are shown in Table 2. It can be seen that, compared to the other three models, the four-factor model achieved the best fit, thus demonstrating good construct validity. This study also carried out further validation of the model fit (Table A1 and Table A2 in Appendix A).

### 4.2. Correlation Analysis Between Variables in This Study

Table 3 shows the mean, standard deviation and correlation coefficients for each variable. As can be seen, all the variables in this study exhibit significant positive correlations, which preliminarily supports the aforementioned hypotheses.

### 4.3. Hypothesis Testing and Discussion

This study used multiple regression analysis to examine the relationships between key variables. Before data analysis, all scale data were standardized, and the results are presented in Table 4. Models 1, 7 and 9 reflect analyses that included only control variables. Model 2 shows that the DL of international students has a significant positive influence on SWB (*β* = 0.425, *p* < 0.001), thus supporting H1.

This study found that the DL of international students has a significant positive direct effect on their SWB. This is consistent with existing research on the use of digital technology and SWB (Feng, 2024; S. Ma et al., 2025b; Pan et al., 2024). DL provides instrumental support for international students as they navigate their studies and daily life in China, helping to reduce negative emotions and enhance life satisfaction. This can be explained through two dimensions: First, DL equips international students with “hard skills” to solve practical problems, enhancing their sense of environmental control and autonomy. For any international student who has just arrived in a foreign country, it is nearly impossible to navigate China’s relatively digitally advanced yet uniquely structured society without adequate DL. Students who are proficient in using online payment systems, for example, can address daily challenges more swiftly and efficiently. This study reveals that DL helps international students overcome survival challenges in their host countries. This sense of self-reliance and competence is a vital source of SWB. It directly reduces anxiety and frustration stemming from unfamiliarity and helplessness, thereby laying a solid foundation for enhanced well-being. Furthermore, DL provides an indispensable channel through which international students can maintain their original social support networks and alleviate feelings of cultural isolation. Past experience shows that maintaining “strong ties” with international students through digital technology provides sustained emotional support and a sense of belonging, which effectively buffers the psychological distress of culture shock. Preserving existing emotional resources through social support facilitated by digital media safeguards emotional and psychological well-being, exerting a stable positive influence on SWB.

Models 8 and 10 respectively demonstrate the direct impact of the DL of international students on PR (Model 8, *β* = 0.760, *p* < 0.001) and PAE (Model 10, *β* = 0.741, *p* < 0.001), indicating a significant effect in these areas. Models 3 and 5, respectively, demonstrate the significant positive impact of PR (Model 3, *β* = 0.493, *p* < 0.001) and PAE (Model 5, *β* = 0.402, *p* < 0.001) on SWB.

Based on Models 2, 3, 4 and 8, the study concludes that PR mediates the relationship between DL and SWB (H2), while Models 2, 5, 6 and 10 show that PAE also mediates this relationship (H3).

To further examine the mediating effect, this study employed the bootstrap method with the Process 4.1 plugin, using 10,000 random samples. As shown in Table 5, PR mediated the relationship between DL and SWB (indirect effect = 0.307, S.E. = 0.046, 95% CI = [0.216, 0.397], excluding 0). Similarly, PAE was found to mediate the relationship between DL and SWB (indirect effect = 0.129, S.E. = 0.053, 95% CI = [0.023, 0.230], excluding 0).

This study identifies PR as a mediating factor between the DL of international students and their SWB (see Figure 2). It also reveals a transformative pathway from “possessing skills” to “shaping character”, and ultimately “enhancing well-being”. These findings partially corroborate the main conclusions of previous studies (Cui et al., 2023; Hedeshi & Ghanizadeh, 2025; Lam & Zhou, 2022; G. Li, 2025). While DL enables international students to navigate daily challenges, PR clarifies how it enables them to “recover” from greater adversity and stress while achieving “growth”. By using digital tools to solve specific problems, international students progressively develop digital competence—the belief that “I can solve problems”. This sense of mastery, gained through sustained digital practice, fosters a more generalized belief in their ability to overcome future challenges. When they encounter academic setbacks or interpersonal conflicts, this heightened self-efficacy motivates them to adopt proactive problem-solving strategies. This process demonstrates how DL transforms international students from passive recipients to active constructors when navigating cross-cultural dilemmas. PR for international students is better able to regulate their emotions under pressure and maintain positive and optimistic attitudes. Consequently, they experience higher overall life satisfaction and more frequent positive experiences, while negative experiences become less frequent.

In addition, this study found that PAE acts as a mediator between international students’ DL and their SWB, as shown in Figure 3. Unlike PR, which is formed in response to external shocks or pressures, PAE reflects the process by which international students use their DL to improve their SWB. This finding is consistent with the results of previous studies (L. Ma, 2025; Prior et al., 2016; Wong et al., 2024; Wu, 2025). Within the context of this research, we propose two key manifestations. First, DL facilitates the transition from “knowledge consumers” to “knowledge creators”. International students with high DL can make their learning journey in China highly interactive and creative. The process of actively exploring, designing, creating and sharing is enjoyable and challenging, and conducive to flow experiences. The sense of accomplishment and creative satisfaction derived from deep engagement represents an intense release of positive emotions, directly contributing to well-being. Secondly, DL enables a shift from being a “peripheral observer” to a “community co-builder”. Upon arriving in a foreign country, many students may initially feel like cultural outsiders. DL can empower them to overcome this by enabling them to proactively build and participate in a meaningful community life. By contributing value through digital media platforms and co-creating community culture with others, students can cultivate a sense of belonging and identity within their host country’s academic and social environment. These profound, positive emotions generate positive experiences and accumulate personal resources, continuously contributing to the SWB of international students.

## 5. Conclusions

### 5.1. Main Findings

In today’s world, where globalization and digitalization are deeply intertwined, DL has become an essential part of international students’ cross-cultural competence. It is no longer merely an optional skill, but rather a guarantee that they will be able to adapt to new environments smoothly, learn efficiently, and integrate successfully. This study presents a dual-path mediation model of the relationship between international students’ DL and their SWB, shedding light on the mechanisms that influence well-being from various angles. Three conclusions are drawn: Firstly, DL is a vital component of international students’ digital competence and directly promotes their SWB. Secondly, DL fosters PR, enabling students to maintain stability and achieve growth amid adversity and thus enhancing their life satisfaction and happiness. Third, DL stimulates PAE, encouraging students to find joy and meaning in daily life and thus enhancing their sense of fulfillment and happiness. Together, these two indirect pathways illustrate how DL can transform from an external skill into an internalized strength, promoting students’ psychological well-being holistically.

### 5.2. Main Significance

This study has the following research significance. First, this study integrates digital literacy research, positive psychology, and cross-cultural adaptation theory to construct an integrative analytical framework for understanding the well-being of international students in the digital age. This framework breaks through the previous limitation of viewing digital literacy primarily as an instrumental skill or a means of academic adaptation, theoretically emphasizes the core role of digital literacy in constructing individual psychological resources and creating positive experiences, and expands the research boundaries of cross-cultural adaptation studies and positive psychology. Second, this study reveals the dual psychological pathways through which digital literacy affects international students’ subjective well-being. Previous studies have mostly adopted an environmental resilience perspective to examine the relationships between climate resilience, urban resilience, and residents’ perceived happiness, emphasizing the impact of the capacity of external environmental systems to cope with shocks on well-being (L. Li et al., 2025a, 2025b). In contrast, this study shifts resilience research to the individual psychological level, focusing on the mediating links of PR and PAE between digital literacy and international students’ subjective well-being. This not only addresses the “black box” of how digital literacy affects well-being but also deepens the understanding of the differentiated roles of individual psychological resources in cross-cultural adaptation. Third, this study provides targeted guidance for Chinese educational institutions and administrative departments. Universities should incorporate digital literacy education into the training system for international students, covering not only basic digital life skills but also strategies for using digital tools for creative learning, community building, psychological adjustment, and stress management. Meanwhile, digital psychological support resources should be strengthened, such as online counseling, international student mutual-aid communities, and cross-cultural digital workshops, so that the digital environment can become a positive space for promoting international students’ psychological adaptation and well-being. In addition, the findings of this study remind educational administrators to value the “psychological empowerment” function of digital literacy rather than treating it merely as technical training. By optimizing campus digital infrastructure, building inclusive digital communities, and providing cross-cultural digital adaptation guidance, it is possible to effectively enhance international students’ PR and PAE, thereby promoting their subjective well-being and the quality of their study abroad experience.

### 5.3. Limitations and Future Directions

This study has the following limitations and future directions. Firstly, this study primarily used survey data and is subject to methodological limitations such as common method bias, sample heterogeneity, and non-probability sampling. Consequently, the findings only partially support and establish causal relationships. In future research, open-ended questions could be added to the scales to inquire about the possible reasons behind respondents’ ratings. Secondly, the cross-sectional nature of the data in this study partially limits causal inference. In terms of research design, future studies could adopt a longitudinal design, measuring and tracking international students’ digital literacy, mediating variables, and subjective well-being at different time points, so as to more rigorously test the dynamic causal relationships among variables and the temporal logic of mediation effects. Quasi-experimental methods such as instrumental variable approaches, propensity score matching, or fixed effects models could also be considered to further control for endogeneity bias. Finally, future research could employ qualitative methods, such as in-depth interviews and narrative analysis, to capture vivid stories of students using digital technology for psychological growth, examine the possibility of cultural intelligence (CQ) or language proficiency as moderating variables, explore whether the impact of digital literacy on well-being differs across international student groups with varying levels of cultural adaptation ability, and even pay attention to the negative effects of digital literacy in the AI era, such as social displacement caused by over-reliance on digital devices and anxiety induced by information overload.

## Figures and Tables

**Figure 1:**
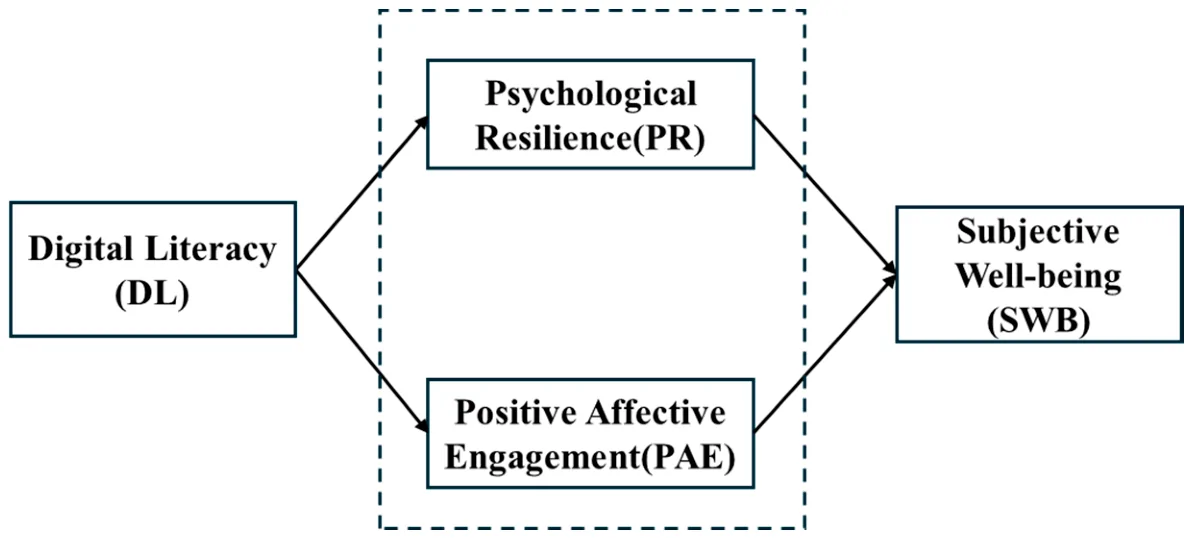
The model of this study.

**Figure 2:**
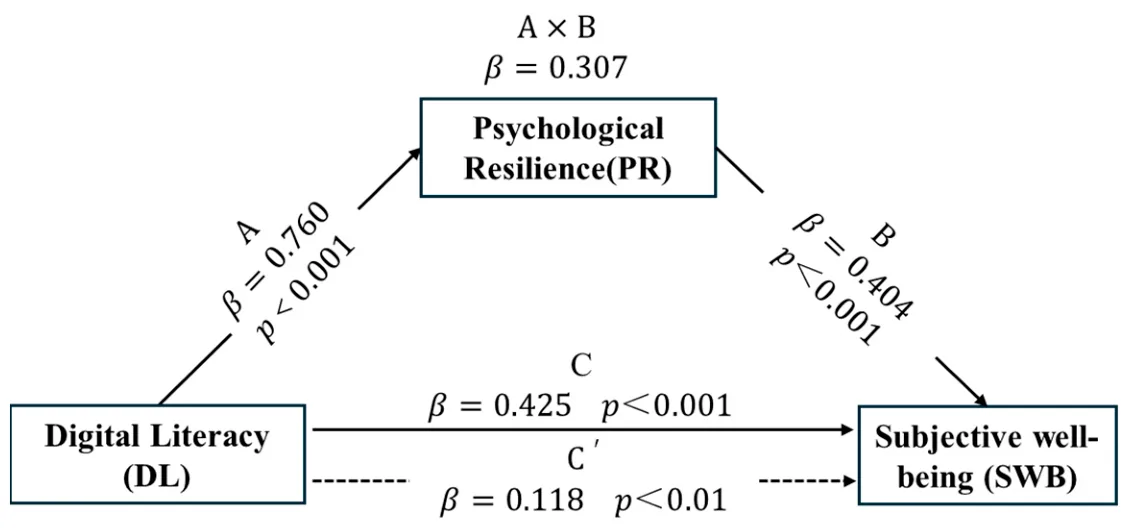
Mechanism of PR.

**Figure 3:**
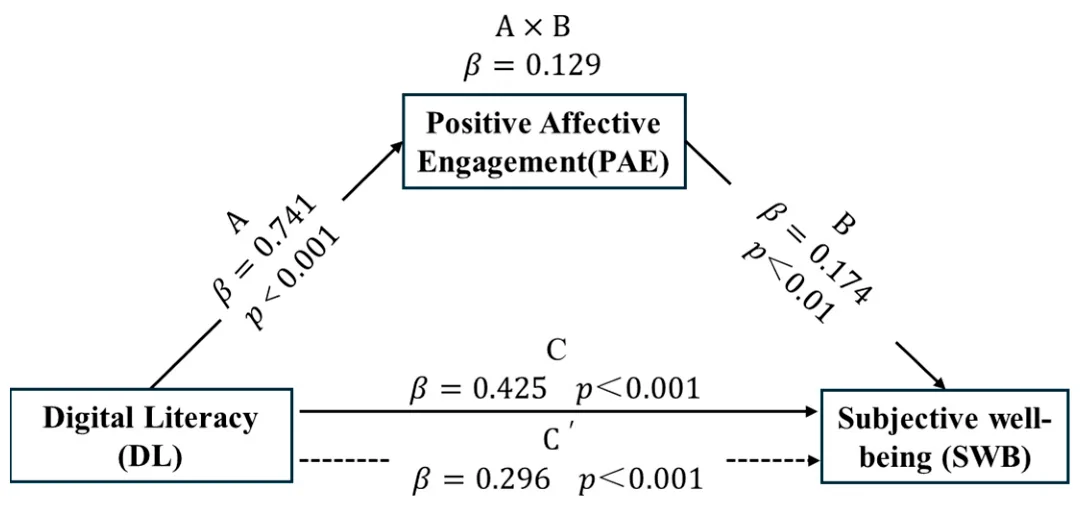
Mechanism of PAE.

**Table 1:** Sample distribution in this study.

Infor.	Opt.	Fre.	Per.	Infor.	Opt.	Fre.	Per.
Gender	Males	191	42	Stay in China (Year)	0–3	327	71.9
	Females	264	58		>3	128	28.1
Educational background	High school or below	39	8.6	Major	Science, engineering, agriculture and medicine	181	39.8
	Undergraduate	296	65.1		Other social sciences, such as philosophy, economics, management and law	63	13.8
	Postgraduate	119	26.3		Chinese-related fields, such as Chinese language, international Chinese education and intercultural education	212	46.4
Age	<18	9	2	Length of Chinese Learning (Year)	<1	85	18.7
	18–22	223	49		1–2	86	19.1
	22–25	116	25.5		3–4	116	25.5
	25–30	66	14.5		5–6	35	7.7
	>30	41	9		>6	132	29.0

**Table 2:** Results of Confirmatory Factor Analysis.

Model	Factors	χ^2^/df	RMSEA	SRMR	CFI	IFI	TLI
Four-factor model	DL, PR, PAE, SWB	2.050	0.048	0.045	0.912	0.912	0.906
Three-factor model	DL, PR + PAE, SWB	2.337	0.054	0.052	0.888	0.889	0.881
Two-factor model	DL, PR + PAE + SWB	2.606	0.059	0.061	0.866	0.866	0.857
Single-factor model	DL+ PR + PAE+SWB	2.867	0.064	0.063	0.844	0.845	0.834

**Table 3:** Correlation Analysis.

	Mean	SD	1	2	3	4	5	6	7	8	9	10
Gender	1.58	0.494	1									
Age	2.80	1.017	−0.316 ***	1								
Study stage	3.22	0.772	−0.206 ***	0.669 ***	1							
Major	1.67	0.705	−0.285 ***	0.196 ***	0.137 **	1						
Cumulative residence time in China	2.08	1.044	−0.115 *	0.214 ***	0.178 ***	0.002	1					
Chinese study time	3.09	1.474	0.114 *	−0.250 ***	−0.221 ***	0.027	0.256 ***	1				
DL	3.83	0.600	−0.092 +	0.154 **	0.207 ***	0.102 *	−0.001	−0.118 **	1			
PR	3.83	0.633	−0.120 *	0.105 *	0.155 **	0.043	−0.033	−0.132 **	0.762 ***	1		
PAE	3.78	0.694	−0.186 ***	0.158 **	0.232 ***	0.101 *	−0.004	−0.173 ***	0.767 ***	0.739 ***	1	
SWB	3.74	0.657	−0.048	0.015	0.082 +	0.029	−0.100 *	−0.072	0.427 ***	0.498 ***	0.400 ***	1

Notes. + *p* < 0.1; * *p* < 0.05; ** *p* < 0.01; *** *p* < 0.001.

**Table 4:** Regression analysis results.

	Model 1	Model 2	Model 3	Model 4	Model 5	Model 6	Model 7	Model 8	Model 9	Model 10
Constant	0.055 (0.340)	0.315 (0.311)	0.030 (0.297)	0.107 (0.299)	0.098 (0.314)	0.255 (0.310)	0.050 (0.330)	0.504 * (0.217)	−0.104 (0.318)	0.334 (0.209)
Gender	−0.050 (0.103)	−0.037 (0.094)	−0.003 (0.090)	−0.008 (0.090)	0.005 (0.096)	−0.017 (0.095)	−0.095 + (0.100)	−0.073 * (0.066)	−0.136 ** (0.096)	−0.115 *** (0.063)
Age	−0.077 (0.065)	−0.074 (0.059)	−0.058 (0.057)	−0.061 (0.057)	−0.049 (0.060)	−0.063 (0.059)	−0.038 (0.063)	−0.033 (0.042)	−0.069 (0.061)	−0.064 (0.040)
Study stage	0.134 * (0.082)	0.056 (0.075)	0.061 (0.072)	0.052 (0.072)	0.046 (0.077)	0.042 (0.075)	0.148 * (0.079)	0.010 (0.053)	0.218 *** (0.076)	0.083 * (0.051)
Major	0.012 (0.070)	−0.018 (0.064)	0.010 (0.061)	0.002 (0.061)	−0.007 (0.065)	−0.017 (0.063)	0.005 (0.068)	−0.050 (0.045)	0.049 (0.065)	−0.005 (0.043)
Cumulative residence time in China	−0.105 * (0.049)	−0.099 * (0.044)	−0.086 + (0.043)	−0.087 + (0.043)	−0.100 * (0.045)	−0.098 * (0.044)	−0.040 (0.047)	−0.028 (0.031)	−0.012 (0.046)	0.000 (0.030)
Chinese study time	−0.030 (0.035)	0.002 (0.032)	0.013 (0.031)	0.0144 (0.031)	0.020 (0.033)	0.014 (0.032)	−0.087 + (0.034)	−0.031 (0.022)	−0.125 * (0.033)	−0.070 * (0.022)
DL		0.425 *** (0.045)		0.118 + (0.066)		0.296 *** (0.069)		0.760 *** (0.032)		0.741 *** (0.031)
PR			0.493 *** (0.043)	0.404 *** (0.065)						
PAE					0.402 *** (0.047)	0.174 ** (0.070)				
R^2^	0.025 +	0.196 ***	0.257 ***	0.263 ***	0.172 ***	0.208 ***	0.043 **	0.589	0.092 ***	0.611 ***

Notes. N = 455, + *p* < 0.1, * *p* < 0.05, ** *p* < 0.01, *** *p* < 0.001.

**Table 5:** Mediating Effect Test.

Hypothesis	Coefficients	S.E.	Bias-Corrected 95%CI		Result
			Lower	Upper	
DL⟶PR⟶SWB	0.307	0.046	0.216	0.397	Supported
DL⟶PAE⟶SWB	0.129	0.053	0.023	0.230	Supported

## Data Availability

The data presented in this study are available upon request from the corresponding author due to restrictions related to participant privacy concerns.

## Appendix A

**Table A1:** Results of HTMT.

	1	2	3	4
DL	-			
PR	0.834	-		
PAE	0.821	0.807	-	
SWB	0.488	0.581	0.457	-

Notes. All values < 0.85.

**Table A2:** Results of Standardized factor loadings, CR and AVE.

	Standardized Factor Loadings	CR	AVE
DL1	0.612	0.933	0.437
DL 2	0.585		
DL 3	0.650		
DL 4	0.689		
DL 5	0.747		
DL 6	0.759		
DL 7	0.707		
DL 8	0.588		
DL 9	0.634		
DL 10	0.579		
DL 11	0.611		
DL 12	0.574		
DL 13	0.705		
DL 14	0.774		
DL 15	0.616		
DL 16	0.672		
DL 17	0.652		
DL 18	0.689		
PR 1	0.708	0.914	0.518
PR 2	0.805		
PR 3	0.584		
PR 4	0.704		
PR 5	0.777		
PR 6	0.790		
PR 7	0.702		
PR 8	0.672		
PR 9	0.749		
PR 10	0.677		
PAE 1	0.774	0.945	0.631
PAE 2	0.804		
PAE 3	0.814		
PAE 4	0.827		
PAE 5	0.743		
PAE 6	0.780		
PAE 7	0.829		
PAE 8	0.810		
PAE 9	0.789		
PAE 10	0.767		
SWB 1	0.755	0.918	0.555
SWB 2	0.666		
SWB 3	0.811		
SWB 4	0.730		
SWB 5	0.719		
SWB 6	0.781		
SWB 7	0.769		
SWB 8	0.755		
SWB 9	0.712		
